# Asthma and COVID-19: a dangerous liaison?

**DOI:** 10.1186/s40733-021-00075-z

**Published:** 2021-07-15

**Authors:** Carlo Lombardi, Federica Gani, Alvise Berti, Pasquale Comberiati, Diego Peroni, Marcello Cottini

**Affiliations:** 1 Departmental Unit of Allergology, Immunology & Pulmonary Diseases, Fondazione Poliambulanza, Brescia, Italy; 2 Departmental Unit of Pneumology & Allergology, Fondazione Poliambulanza Istituto Ospedaliero, Via Bissolati, 57, 25100 Brescia, Italy; 3 Allergy Outpatients Clinic, Turin, Italy; 4 Ospedale Santa Chiara and Department of Cellular, Computational and Integrative Biology (CIBIO), University of Trento, Trento, Italy; 5 Thoracic Disease Research Unit, Mayo Clinic, Rochester, MN USA; 6 Department of Clinical and Experimental Medicine, Section of Pediatrics, University of Pisa, Pisa, Italy; 7 Department of Clinical Immunology and Allergology, I.M. Sechenov First Moscow State Medical University, Moscow, Russia; 8 Allergy and Pneumology Outpatient Clinic, Bergamo, Italy

**Keywords:** SARS-COV-2, COVID-19, Asthma, Phenotypes, Therapy, Biological agents, Biologics, Immunotherapy

## Abstract

The coronavirus disease 2019 (COVID-19) pandemic, caused by the new severe acute respiratory syndrome coronavirus 2 (SARS-CoV-2), provoked the most striking international public health crisis of our time. COVID-19 can cause a range of breathing problems, from mild to critical, with potential evolution to respiratory failure and acute respiratory distress syndrome. Elderly adults and those affected with chronic cardiovascular, metabolic, and respiratory conditions carry a higher risk of severe COVID-19. Given the global burden of asthma, there are well-founded concerns that the relationship between COVID-19 and asthma could represent a “dangerous liaison”.

Here we aim to review the latest evidence on the links between asthma and COVID-19 and provide reasoned answers to current concerns, such as the risk of developing SARS-CoV-2 infection and/or severe COVID-19 stratified by asthmatic patients, the contribution of type-2 vs. non-type-2 asthma and asthma-COPD overlap to the risk of COVID-19 development. We also address the potential role of both standard anti-inflammatory asthma therapies and new biological agents for severe asthma, such as mepolizumab, reslizumab, and benralizumab, on the susceptibility to SARS-CoV-2 infection and severe COVID-19 outcomes.

## Introduction

The coronavirus disease 2019 (COVID-19) pandemic, caused by severe acute respiratory syndrome coronavirus 2 (SARS-CoV-2), provoked the most striking global health crisis of the last century [1, 2]*.*

COVID-19 can cause a range of breathing problems, from mild to critical, with older adults and people who have chronic comorbidities, such as hypertension, chronic obstructive pulmonary disease (COPD), obesity, heart disease, cancer, and diabetes, carrying a higher risk of severe symptoms [3]*.* Given the impact of SARS-CoV2 infection on the respiratory system on one side and the epidemiological burden of bronchial asthma worldwide on the other side, there are well-founded concerns that the relationship between COVID-19 and asthma could become a “dangerous liaison”.

In this regard, the relationship between asthma and COVID-19 needs to be better defined. This wide aspect could be further dissected in several questions that need to be addressed: 1) are asthmatics at increased risk of SARS-CoV-2 infection and/or severe COVID-19? 2) could different asthma endotypes (type 2 asthma vs. non-type 2 asthma) carry a different risk profile in terms of SARS-CoV-2 infection, COVID-19 development, and progression to severe disease outcomes? 3) if so, could type-2 asthma provide any protection against SARS-CoV-2 infection and/or severe COVID-19? 4) could smoking, asthma-COPD overlap (ACO), or obesity increase the risk of SARS-CoV-2 infection and/or severe COVID-19 in asthmatics? 5) could inhaled corticosteroid and bronchodilator therapy for asthma and new biological agents targeting type 2 inflammation, such as mepolizumab, reslizumab, and benralizumab, affect the susceptibility to SARS-CoV-2 infection and/or the risk of severe COVID-19? The ambitious aim of this review is to collect the latest evidence regarding the intricate relationship between asthma and COVID-19 and provide reasoned answers to the questions above.

### Asthma and non-SARS CoV-2 viral infections

Several factors have been associated with increased risk for COVID-19 severity and mortality, such as older age, male sex, comorbidities, and metabolic abnormalities [3, 4]. Early in the pandemic, asthma was also suggested as a risk factor for COVID-19 [5].

It seems plausible to think that a patient with asthma would be at increased risk of SARS-CoV-2 infection and more serious manifestations of COVID-19 because asthmatics normally carry an increased susceptibility to common viral respiratory infections [6], partly due to a deficient and delayed innate antiviral immune response. Asthmatic patients also show an increased frequency and severity of lower respiratory tract infections compared to healthy individuals [7]. Moreover, viral respiratory tract infections are a major trigger of asthma exacerbations in both children and adults. In particular, human rhinovirus is detected in 76% of wheezing children and 83% of adult exacerbations [8–10]. The influenza virus can also favor asthma exacerbations, while other viruses, such as coronavirus, adenovirus, parainfluenza virus, metapneumovirus and bocaviruses, seem to be potential triggers of acute asthma but to a lesser extent [11].

Environmental exposures and allergies can further boost the risk of virus-induced exacerbations [12].

Impaired innate immune responses have been observed in asthmatics. A high proportion of patients with asthma and atopic disease have a predisposition to produce lower levels of type I interferon (INF) ro other cytokines upon viral respiratory infections [13–15].

Through different mechanisms, Type 2 inflammation may have an inhibitory effect on the induction of type I interferon [16]. Intriguingly, defective production of IFNs by plasmacytoid dendritic cells (pDCs) and epithelial cells have been described in severe atopic patients [17] with a consequent delayed and inefficient antiviral defense. In this context, a cross-regulation mechanism between FceRI and TLRs in certain cell types such as pDCs has been described, which may explain why the crosslinking of IgE bound to FceRI by allergens may result in a reduced TLR expression and ultimately in a decreased capacity to secrete type I interferons for viral defense [16, 18]. Asthmatic patients are known to be at greater risk of influenza-related complications as previous studies have shown that asthma is common among patients hospitalized with influenza [19, 20]. During the Swine Flu pandemic, asthma was an undisputed risk factor associated with hospitalization, affecting 10–20% of the hospitalized populations worldwide [21] and, among patients hospitalized in the United States during April–June 2009, asthma was the most reported underlying chronic medical condition, affecting 28% of patients [22]. Asthmatic patients compared with non-asthmatic subjects were almost twice as likely to have pneumonia (50% vs. 27%) and required care in the intensive care unit (ICU) (33% vs. 19%) [23]. Finally, the risk for community-acquired bacterial and viral pneumonia has been estimated to be at least 2-fold in asthmatic patients compared with healthy control subjects [24].

### Susceptibility of patients with asthma to COVID-19 infection

In the early stage of the pandemic, asthma was inconsistently mentioned among the significant clinical risk factors for SARS-CoV-2 infection in studies from China [4, 25–27] and Italy [28–30]. Studies from Russia, Saudi Arabia, Brazil, and India also reported lower rates of asthma among patients with COVID-19 [31–33]. Instead, studies from the USA and the UK reported that comorbidity rates of asthma in patients with COVID-19 were similar or higher than those in the local population [34–41]. Recently, Broadhurst et al. performed a focused literature review among patients hospitalized for COVID-19 infection; their findings suggest that asthma prevalence appears to be similar to population asthma prevalence and significantly lower than asthma prevalence among patients hospitalized for influenza [42]. Kalyanaraman et al. reviewed the electronic health records (New York City’s public hospital system) of all patients who received a SARS-CoV-2 test and showed that asthma was not associated with testing positive [43]. A systematic review and meta-analysis of 131 studies from 39 countries (410,382 patients) reported asthma prevalence in adult or all-age-group patients with COVID-19. The regional asthma comorbidity rates were estimated as follows: East Asia and the Pacific, 2.2%; Europe, 6.4%; Latin America and the Caribbean, 3.5%; the Middle East and North Africa, 4.9%; North America, 10.2% [44]. Very recently, Terry et al. performed a systematic review and meta-analysis of 150 studies and did not find clear evidence of increased risk of COVID-19 diagnosis in asthmatics [45].

In conclusion, there is great variability in the prevalence of asthma among patients with COVID-19 in different countries; in most countries patients with asthma were not reported with higher, but rather similar or lower rates of COVID-19 infection, compared with the general population in the corresponding area, probably due to multiple factors including a low proportion of non-type 2 phenotypes [38]. Indeed, a Korean nationwide cohort showed that patients with non-allergic asthma had a greater risk of SARS-CoV-2 test positivity than patients with allergic asthma [46].

### Risk of morbidity and mortality in patients with asthma and COVID-19

Results are heterogeneous when examining the association between asthma and severity of COVID-19. A study that analysed the UK Biobank data (493,000 patients) showed that adults with asthma had a higher risk of severe COVID-19 [41], and in a Korean nationwide cohort, asthma confers a greater risk of susceptibility to SARS-CoV-2 infection and severe clinical outcomes of COVID-19 [46]. An interesting aspect to note is that, in both studies, the higher risk of severe COVID-19 was driven by the increased risk in non-allergic asthma patients. In contrast, several studies found no statistically significant association between asthma and mortality or risk of intubation/mechanical ventilation in patients with COVID-19. In the International Severe Acute Respiratory and Emerging Infections Consortium (ISARIC) World Health Organization (WHO) Clinical Characterization Protocol UK study, despite a prevalence of 14.5%, asthma was not associated with an increased risk of ICU admission, mechanical ventilation, or death [39]*.* Broadhurst et al., using a cross-sectional analysis of patients with COVID-19 admitted to the University of Colorado Hospital, showed that asthma does not appear to be an independent risk factor for intubation among hospitalized patients with COVID-19, even after adjusting for well-known risk factors for severity [42]. Two independent studies [47, 48] similarly demonstrated that patients with COVID-19 comorbid with chronic obstructive pulmonary disease or diabetes tended to be more severe, whereas those comorbid with asthma did not. In a recent matched cohort study conducted in Boston among patients hospitalized for COVID-19, asthma was not associated with an increased risk of ICU admission, hospitalization, mechanical ventilation, or death compared with inpatient comparators matched by age, sex, and date of positive SARS-CoV-2 test [49, 50]. Moreover, in three studies from New York, among hospitalized patients with severe COVID-19, asthma diagnosis was not associated with worse outcomes and mortality [43, 51, 52]. Calmes et al. collected data from 596 adult patients hospitalized for SARS-CoV2 infection. The multivariate analysis showed that asthma was not an independent risk factor for ICU admission or death [53]. Patients with COPD, but not asthma, have a slightly increased risk of severe outcomes of COVID-19 compared with patients without obstructive lung disease [54]. Murillo-Zamora et al. conducted a nationwide, retrospective cohort study in Mexico in which data from 66,123 individuals were analyzed. Reduced risk of a fatal outcome was observed among patients with asthma history [55]. In a systematic review and meta-analysis of 131 studies from 39 countries (410,382 patients), no significant difference in asthma prevalence was found between hospitalized and non-hospitalized, severe and non-severe, ICU and non-ICU, dead and survived, intubated/mechanically ventilated and non-intubated/mechanically ventilated patients with COVID-19.

Patients with asthma have a lower risk of death compared with patients without asthma [44]. The overall findings of another recent meta-analysis (587,280 patients) suggest that people with asthma have a lower risk than those without asthma of acquiring COVID-19 and have similar clinical outcomes [56]. Finally, Terry et al. review the literature related to the role of asthma on COVID-19 outcomes: the results of this meta-analysis do not provide clear evidence of increased risk of COVID-19, hospitalization, or severity, due to asthma [45]. Moreover, the authors reported a significant 18% reduction in risk of mortality in asthma patients with COVID-19 compared to non-asthmatic patients. This finding is more robust due to the adjustment for major confounding factors, and provides reassurance to asthma sufferers, and those responsible for their care.

Using big data analytics and artificial intelligence through the SAVANA Manager® clinical platform (71,182 patients with asthma), Izquierdo et al. analyzed clinical data from patients with asthma [57]. The manifestation of the disease in this clinical population was not particularly severe, with a low rate of hospital admissions. The increased risk for hospitalization due to COVID-19 in patients with asthma was largely associated with age and related comorbidities. Moreover, in a recent study from France, among 768 hospitalized patients (37 (4.8%) with a history of asthma) worse outcomes were observed mainly in asthmatics with major comorbidities [58]. A study from the UK examined the association between different phenotypes of asthma and COVID-19 infection. Interestingly, the risk was mostly related to non-allergic asthma [59]. Unfortunately, there is still little information about asthma phenotypes in patients with COVID-19, but it is possible to speculate that different asthma endotypes may also have a differential impact on the progression and severity of COVID-19 [38, 41, 59]. These data need further examination in prospective large cohort studies.

### SARS-CoV-2 and asthma exacerbations

Quite surprisingly, on April 8, 2020, Morbidity and Mortality Weekly Report (MMWR) published a report of 1482 patients hospitalized for COVID-19 in the USA, it was mentioned that wheezing was present in only about 7% of the patients for whom data were available on underlying conditions, which is less than the prevalence rate of about 10% of asthma in the general population [35]. This report suggests that SARS-CoV-2 rarely induces asthma exacerbations during hospitalization for COVID-19. Also in other studies, few patients were hospitalized for a COVID-19-related asthma exacerbation during the outbreak and very few developed an asthma attack during hospitalization [58, 60]. These data thus reflect a striking difference from previous respiratory viral pandemics, most recently the H1N1/A outbreak in 2009, also because viral infections, including other types of non-SARS coronaviruses, are the main cause of asthma exacerbations.

### Severe asthma and COVID-19

A minority of patients with asthma (5–10%) have uncontrolled or partially controlled asthma despite intensive treatment. One would expect increased vulnerability to SARS-CoV-2 infection in these patients, but scarce data are so far available to confirm this hypothesis. Despite the lack of sufficient evidence, the Center of Disease Control (CDC) in the USA issued warnings that patients with moderate-to-severe asthma may be at increased risk of contracting COVID-19 and suffer severe outcomes from the disease [61]. A database from the UK looking at electronic health records (EHR) of 17 million patients with COVID-19 showed an association between severe asthma and increased risk of death for hospitalized patients after adjustment for sex and race, but not for all comorbidities. The hazard ratio was higher for patients with asthma with documented recent oral corticosteroid use, which is a marker of the severity of asthma [62]. More recently, in the same cohort, Schultze et al. found that patients with asthma taking high-dose inhaled corticosteroids (ICSs) had a 55% increased risk of death from COVID-19, and which was interpreted as being due to confounding by disease severity [63]. Lee et al. selected 7272 adult COVID-19 patients (686 with asthma) from the Korean Health Insurance Review and Assessment COVID-19 database. Asthma was not a significant risk factor for respiratory failure or mortality among all COVID-19 patients. However, a history of acute exacerbation (OR = 2.63, *p* = 0.043) was a significant risk factor for death among COVID-19 patients with asthma [64]. In anotherstudy, asthma severity was not an independent factor for poor clinical outcomes of COVID-19, but patients with step 5 asthma had a prolonged in-hospital stay duration than those with step 1 asthma in both univariate and multivariate analyses [65]. A large COVID-19 hospitalized Italian population showed that patients with worse COVID-19 outcome (death/need for ventilation vs. discharge at home without requiring invasive procedures) suffered from more severe asthma (GINA 4/5, 43 vs. 14%, *p* = 0.040) [66]. Although biologic-treated patients with asthma typically present with the most severe manifestations of the disease, these studies showed that the number of COVID-19-related admissions and mortality in these patients was strikingly low [67–74]. Treatment with biologicals for severe asthma also seems to have no significant effect on the outcome of COVID-19.

In conclusion, it is now well-recognized that older age, obesity, cardiovascular diseases, and diabetes are risk factors of poor COVID-19 outcome. What is not yet clear is whether chronic respiratory diseases like asthma are also to be included as risk factors. The many studies that have addressed this question show discrepant results and point towards numerous factors that may play a role in the susceptibility and severity of COVID 19 in asthma patients. These include the severity of asthma itself, the asthma phenotypes/endotypes, asthma medications and co-morbidities. The reported incidences of severe COVID-19 cases among asthma patients are not determined by patient-related factors alone. Moreover, local factors (testing policies or shielding advice, such as the case of the older patients or those with co-morbidities like asthma better protected themselves) and the diagnostic methods to identify asthma and COVID-19 can play an important role [75].

### Why might asthma protect against poor outcomes in COVID-19?

**Fig. 1:**
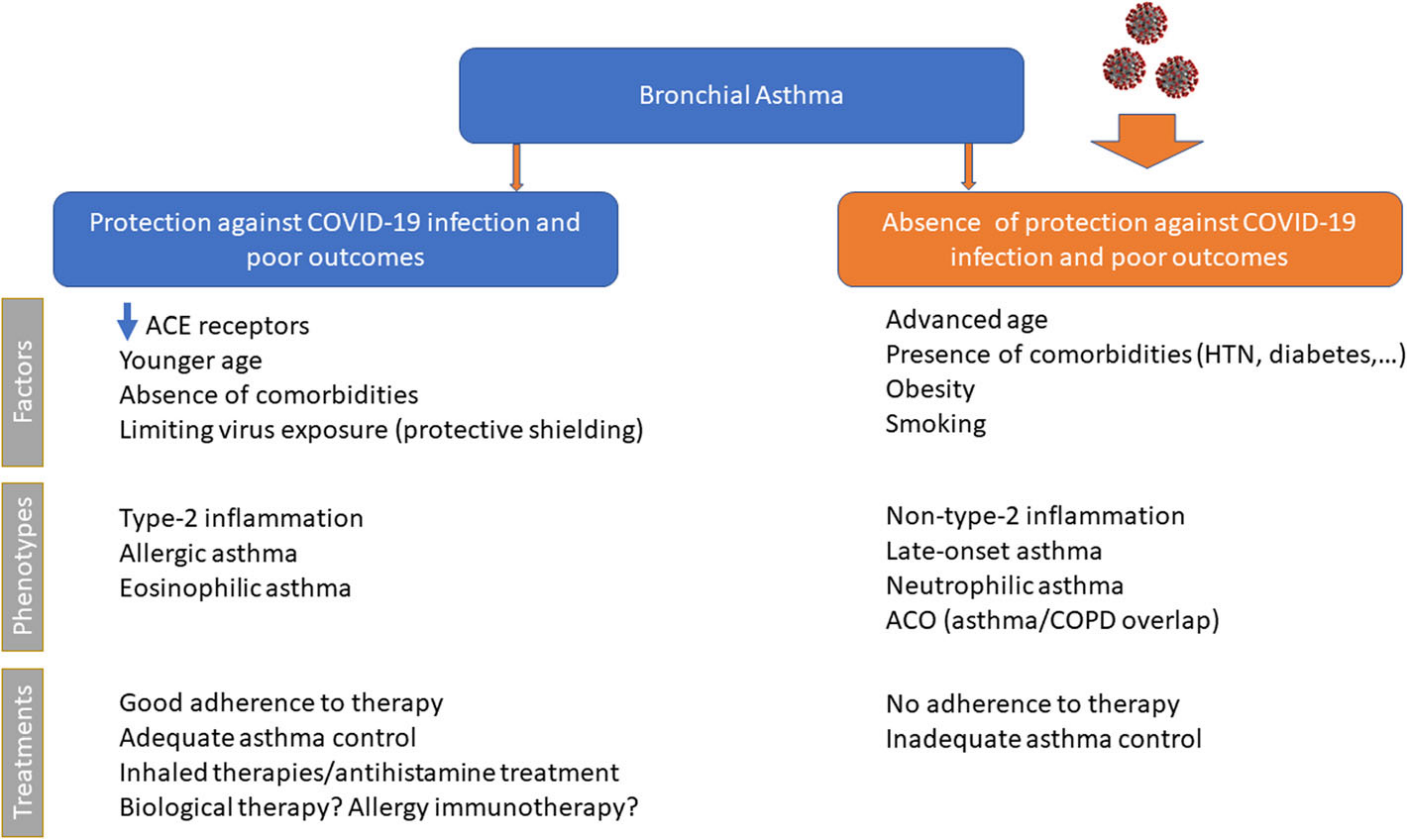
Possible mechanisms by which asthma might protect against poor outcomes of COVID-19

Several studies have suggested possible non-harmful or protective effects of asthma on the clinical outcomes of COVID-19. Asthma might protect against poor outcomes in COVID-19 due to several possible mechanisms (Fig. 1), including altered viral entry receptor expression, chronic type-2 inflammation, younger age and/or absence of comorbidities, reduced exposure due to shielding, increased adherence to therapy and ICS use [76].

#### ACE2 receptor

The lack of susceptibility to COVID-19 in patients with pre-existing allergic asthma seems to be in contrast with the established link between these chronic respiratory conditions and susceptibility to common respiratory viruses, especially rhinoviruses [10]. However, rhinovirus uses the intercellular adhesion molecule 1 (ICAM-1) molecule as an entrance into respiratory epithelial cells, which is overexpressed in allergic airways as a marker of allergic inflammation [77]. In contrast, COVID-19 uses another host cell receptor abundantly present in the oral mucosa and within the (healthy) airways, i.e., the angiotensin-converting enzyme2 (ACE2) [78], which plays a crucial role in the disease development and associated lung injury [79]. Cofactors facilitating SARS-CoV-2 infectivity are transmembrane peptidase serine 2 (TMPRSS2), which cleaves the SARS-CoV-2 spike protein, and possibly protease furin [80]. Peters et al. [81] analyzed gene expression for ACE2 and TMPRSS2, and ICAM-1 (rhinovirus receptor as a comparator) in sputum cells from 330 participants in the Severe Asthma Research Program-3 and 79 healthy control subjects. Among patients with asthma, male sex, African American race, and history of diabetes mellitus were associated with higher expression of ACE2 and TMPRSS2. The use of ICSs was associated with lower expression of ACE2 and TMPRSS2. The asthma endotype is especially important, as cytokines can modify ACE expression. Song et al. [79] found that the mRNA expression levels of ACE2 in bronchial epithelial cells were significantly downregulated in allergic asthmatics compared to healthy controls. A lower expression of ACE2 has been described in airway cells of patients with respiratory allergy and/or asthma, while non-allergic asthma was not associated with ACE2 expression [82]. Furthermore, Kimura et al. reported that IL-13 exposure reduced ACE2 expression in airway epithelial cells from patients with asthma and atopy [83]. These findings suggest that patients with allergic asthma might be protected from COVID-19 because of the low expression of ACE2 in their epithelial cells [84]. By analyzing ACE2 gene expression in bronchial epithelial cells in asthmatic patients with different endotypes, Camiolo et al. identified a positive correlation between ACE2 expression and scores of T1 gene expression and a negative correlation between ACE2 expression and scores of Type-2 gene expression [85]. Kermani et al. [86] examined microarray mRNA expression of ACE2, TMPRSS2 and FURIN in sputum, bronchial brushing, and bronchial biopsies of the European U-BIOPRED cohort. Sputum FURIN expression levels were strongly associated with neutrophilic inflammation and with inflammasome activation. This might indicate the potential for a greater morbidity and mortality outcome from SARS-CoV-2 infection in non-type-2, neutrophilic severe asthma [79]. IL-17, which is produced by Th17 cells and type 3 ILCs, can stimulate neutrophilic airway inflammation and can upregulate ACE2 expression [83]. In a study from US observations in bronchial brush airway, epithelial cells identified a positive correlation between ACE2 gene expression and a previously described IL-17-dependent gene expression signature, with an inverse association with TH2 gene expression [87]. Smoking can also modulate ACE2 expression in the lungs of asthmatics. In an experimental model of smoke-induced acute respiratory distress, a Th17/neutrophilic syndrome, ACE2 was upregulated [88]. In addition, cytokine release from smoking-associated lung injury induces upregulation of ACE2 in the lungs [89]. In conclusion, these data strongly suggest an association between asthma endotypes and ACE2 gene expression.

#### Inflammatory endotypes and COVID-19

Asthma is a complex and very heterogeneous respiratory disease, with differences from patient to patient in causes and drivers, the severity of symptoms, type and degree of inflammation, and response to treatment. The identification of the different patient groups and the different underlying pathophysiological mechanisms in asthmatic patients appears to be very important in assessing the relationships between asthma and COVID-19. A type 2 inflammation is evident in more than 50% of those with a formal asthma diagnosis [90] and is typically characterized by activation of proinflammatory cytokines including interleukins (IL)-4, − 5, and − 13, manifesting as the type 2 endotype with raised levels of immunoglobulin E (IgE), eosinophils, and fractional exhaled nitric oxide (FeNO), and dysfunction of epithelial or epidermal barriers [91]*.* The Type 2-high endotype can have either allergic or non-allergic underpinnings and is typically characterized by some degree of eosinophilic airway inflammation, while the neutrophilic or pauci-granulocytic airway inflammation is associated with the Type-2-low endotype [92]. Several studies supporting the hypothesis that type 2 asthma does not represent a major risk factor for increased COVID-19 severity. A recent study showed that patients suffering from different asthma endotypes (type 2 asthma vs. non-type 2 asthma) present with a different risk profile in terms of SARS-CoV-2 infection, development of COVID-19, and progression to severe COVID-19 outcomes [38]. In a study from the USA, atopy was associated with significantly lower odds of hospitalization for COVID-19 [93]. Moreover, non-allergic asthma was associated with prolonged intubation time. A large population-based cohort study demonstrated that adults with asthma had a higher risk of severe COVID-19, which was driven by the increased risk in patients with non-allergic asthma [41]*.* Again, in a Korean nationwide cohort, patients with non-allergic asthma had a greater risk of SARS-CoV-2 test positivity and worse clinical outcomes of COVID-19 than patients with allergic asthma [46]. Finally, in a retrospective study on patients with SARS-CoV-2-induced pneumonia, hospitalized in several Italian hospitals, atopic subjects showed a much lower occurrence of severe or very severe COVID-19 pneumonia (33.3% vs. 67.7%, *p* < 0.0001) [94].

The second major subgroup of asthma is non-type 2 asthma, which contains a heterogeneous group of endotypes and phenotypes, such as obesity-induced asthma, smoking-related asthma, etc. Non-eosinophilic asthma is generally associated with the absence of eosinophils and activation of non-predominant type 2 immunological pathways [95]. Non-type 2 asthma involves greater Th1/Th17 activity than does atopic asthma, when bronchial epithelial cells release IL-33, IL-6, IL-23, IFNγ, and tumor necrosis factor-α in response to various irritants, resulting in neutrophilic airway inflammation [96]. The major mechanism leading to a non-type 2 response is thought to result from an irregular innate immune response, including intrinsic neutrophil abnormalities and activation of the IL-17-mediated pathway. IL-17, which is produced by Th17 cells and type 3 ILCs, can stimulate neutrophilic airway inflammation. Peters et al. reported that systemic IL-6 inflammation (a biomarker of non-type 2 asthma) occurs in a large subgroup of patients with asthma, most of whom are older and obese [97], and reported that systemic IL-6 inflammation as a biomarker for patients who have both metabolic dysfunction and severe asthma. Non-type 2 asthma is more frequent in women than in men, particularly those over 35 years of age [98]. Women with asthma have a combination of phenotypic heterogeneities, including a Th1 immunological skewness, a predisposition towards more severe asthma [99], structural lung parenchymal differences, and hormonal differences, which might increase their susceptibility to severe COVID-19 requiring hospitalization. The increased prevalence of non-atopic asthma in women might be related to distinct underlying causes of asthma, including obesity. Obese women have a disproportionate incidence and severity of asthma because of increased leptin concentrations, which promote inflammatory Th1 pathways [98–100]. Moreover, a study demonstrated an increase in neutrophilic airway inflammation in obese asthma, compared to obese healthy controls, and this relationship was significant only in females with asthma [101]. A study by Atkins and colleagues established female sex as an independent risk factor for SARS-COV2-related hospitalizations among patients with asthma in the UK [59]. This study and three others from Paris, France, Illinois, USA, and New York, USA, report that 56–71% of patients with asthma hospitalized for COVID-19 were women [50, 51, 58]. Besides obesity, pauci-granulocytic/neutrophilic asthma has been associated with environmental and/or host factors, in particular with smoking cigarettes. Cigarette smoke can damage the epithelium directly and has been associated with non-eosinophilic airway inflammation compared to never smokers with asthma. Through direct activation of macrophages, these cells produce inflammatory molecules, tissue proteases like MMP, IL-8, and other chemokines involved in the mobilization and prolonged survival of neutrophils in the lung tissue, while producing less IL-10, which leads to a non-type 2 pattern with a reduced B-cell number and lower levels of IL-4 and IL-5 [102]. Smokers and COPD patients presented an increase in COVID-19-associated inflammatory markers during the disease course in comparison to non-smokers and former smokers. Current reviews indicate that nicotine exposure is linked to cardiopulmonary vulnerability to COVID-19 and tobacco use can be a potential risk factor for not only getting the viral infection but also its severe manifestations [103–105]. Alberca et al. recently demonstrated that smoking and COPD are risk factors for severe COVID-19 with possible implications for the ongoing pandemic [106]. Together, the pulmonary and systemic effects of cigarette smoking could further potentiate SARS-CoV-2-induced endothelial dysfunction and systemic inflammation in asthmatic patients. Patients with COPD, but not asthma, have an increased risk of severe outcomes of COVID-19 compared with patients without obstructive lung disease [54]. Moreover, in the ISARIC WHO Clinical Characterization Protocol UK study, COPD was associated, unlike asthma, with an increased risk of ICU admission, mechanical ventilation, or death [39]*.* Pathological features of both asthma and COPD coexist in some patients and this is termed ACO [107]. Presently, this patient group is estimated to encompass 11.1–61.0% of the 339 million patients with asthma and 4.2–66.0% of the 252 million patients with COPD, worldwide [108]. Various cardinal features of asthma (reversible airflow limitation and eosinophilic/type-2 inflammation) and COPD (irreversible airflow limitation and neutrophilic/type-1 inflammation) frequently coalesce in patients with ACO [109]. A population-based prospective cohort study analyzed data from the UK Biobank. Participants with asthma, compared with those without, had a significantly higher risk of severe COVID-19 (odds ratio [OR], 1.44). These findings were driven by the significant association of non-allergic asthma with severe COVID-19 (adjusted OR, 1.48). In the stratified analysis by coexisting COPD (*n* = 7815), the significant association persisted in both strata, with a larger magnitude in asthma with COPD (adjusted OR, 1.82) [41]. Therefore, it seems obvious to think that patients with asthma-COPD overlap would be at increased risk of SARS-CoV-2 infection and a more serious clinical picture of COVID-19. The Th17/neutrophilic endotype of asthma in smokers, ACO and obese patients might be exacerbated by the systemic inflammatory response of SARS-CoV-2 infection, which is similarly driven by Th1-related cytokines, including IFNγ, IL-6, MCP1, IP10, and IL-1β [110].

#### Eosinophilic inflammation

Further, the role of eosinophils, foes in asthma but possibly friends in COVID-19 infection, needs to be established [111]. Previous experimental studies indicated a potential role of eosinophils in promoting viral clearance and antiviral host defense [112]. The eosinophils are reduced in peripheral blood of SARS-CoV-2-infected patients [113]; therefore, it is tempting to speculate that increased numbers of eosinophils in the airways of asthmatic patients might be protective against the exaggerated inflammatory responses of the severe COVID-19 phenotype [111]. Patients with the type-2 low asthma endotype who have low eosinophils might be more prone to more severe COVID-19 outcomes, in the same way as in non-allergic asthma. Severe COVID-19 occurring in susceptible individuals may be associated with cytokine-mediated hyper-inflammation and associated coagulopathy with multisystem involvement and death [114]. Markers of worsening disease include hypoxemia, lymphopenia, thrombocytopenia, and raised levels of IL-6, C reactive protein, ferritin, lactate dehydrogenase, and D-dimers. Eosinopenia may also be part of the overall cytopenic process in the early phase of severe COVID-19, with the later resolution of eosinophil counts being associated with clinical recovery [115]. Peripheral blood eosinophil counts may, therefore, be an effective and efficient indicator in diagnosis, evaluation, monitoring and prognosis of COVID-19 patients [116]. Recently, Ferastraoaru et al. [117] retrospectively identified 737 asthma patients with COVID-19 seen in the emergency department (ED). In asthmatics, pre-existing eosinophilia (AEC ≥ 150 cells/mL) was protective from COVID-19 associated hospital-admission, and development of eosinophilia (AEC ≥150 cells/ mL) during hospitalization was associated with decreased mortality. According to the authors, having a Type 2-asthma endotype might be an important predictor for reduced COVID-19 morbidity and mortality which should be further explored in prospective and mechanistic studies. Based on the current evidence and clinical observations, it could be suggested that Type-2 airway disease associated with eosinophilic infiltration and down-regulation of ACE2 does not represent a risk factor for COVID19 infection [114]. Instead, Type 2-low asthmatics demonstrated characteristics corresponding to risk factors for severe COVID-19, including obesity and history of smoking and hypertension. This group of asthmatics has a different inflammatory profile, and due to the chronic sub-clinical inflammation associated with metabolic dysregulation, there is circumstantial evidence that the immune system is already (pre-) programmed to develop hyper-inflammation in the context of a cytokine storm in association with COVID-19. Taken together, although type-2 asthma appears to be a protective factor for COVID-19, the associations between different phenotypic and endotypic asthma and COVID-19 remain to be better defined.

### Younger age and/or absence of comorbidities

Susceptibility and severity of COVID-19 infection increase with age [118], therefore, age is an important confounder in the assessment of the risk of contracting severe COVID-19. Izquierdo et al. showed that asthma patients without COVID-19 were younger and more likely to have eczema and rhinitis, while those with COVID-19 were older and more likely to have co-morbidities such as hypertension and diabetes [57]. In addition, children and young adults with asthma manifest Type-2 high airway inflammation that is driven predominantly by allergy, IL-4 and IL13. In comparison, non-type 2 asthma is more frequent in older adults, particularly in women over 35 [98] and presents with a different risk profile in terms of comorbidities, SARS-CoV-2 infection, development of COVID-19 and progression to severe COVID-19 outcomes [38]. Expression of ACE2, the co-receptor for SARS-CoV-2, varies with age [81]. Because Type-2 asthma sufferers tend to be younger than those with other comorbidities, the age factor probably explains why patients with asthma may not be at greater risk. However, to better address this question, age-adjusted models need to be formulated.

### Protective shielding limiting virus exposure

Behavioral factors are likely to be important. Protective shielding for at-risk groups, including those with asthma, has been widely advocated by international guidelines. Reduced exposure to SARS-CoV-2 amongst patients with asthma may therefore be contributing to the low prevalence of asthma reported in hospitalized cohorts [76]. Government policies to limit the spread of the pandemic have also led to reductions in air pollution, which increases the severity of virus-induced asthma exacerbations [119]. Nationwide preventive measures for COVID-19 in Japan were associated with a sharp drop in hospitalizations for asthma as a secondary effect [120].

### Adherence to therapy

As hospitalizations for asthma are considered a consequence of poor asthma control [121], these findings suggest that asthma was better controlled during this outbreak. Initial concerns that the COVID-19 spread might increase asthma attacks may have encouraged preventive behaviors among people with asthma and their families, including quitting smoking, cleaning their rooms more frequently to remove allergens, and better adherence to preventive medications. Patients with asthma are advised to closely adhere to their prescribed inhaler medication therapy due to the COVID-19 pandemic. In an interesting study, a cohort of 7000 patients had electronic monitoring of their controller and rescue inhaler use during the pandemic, and increased adherence of 15% was found in the controller medication use [122]. According to the results of a cross-sectional study from Mexico, male sex, active smoking, and the belief that COVID-19 was not more severe in asthma sufferers seemed to favor non-adherence to COVID-19 prevention measures [123]. It is therefore important that health professionals and patients with asthma maintain constant communication regarding the measures that patients must comply with to prevent COVID-19 and the timely use of medications to control their chronic disease.

## Anti-asthmatic therapies in the context of COVID-19: protective or favorable role?

### Inhaled antiasthmatic therapies and COVID-19

It is likely that maintenance of ICSs can also confer protection, but there is no evidence of the benefits or harm of ICSs in COVID-19 [124]*.* Several key questions arise. Are asthmatics, or some phenotypes, at increased risk of developing COVID-19? Do ICSs modify this risk, either increasing or decreasing it? Do ICSs influence the course of COVID-19? Epidemiological studies of COVID-19 must include detailed information on asthma comorbidity and prior medication to help answer these questions [125]*.* Today, there is no evidence of an association of increased risk of COVID-19 infection in asthmatic patients regularly taking ICSs [126]. Asthma exacerbations have been markedly reduced especially in children during the COVID-19 pandemic, which may not only be due to a decrease in exposure to triggers, i.e. air pollutants and aeroallergens, but may also be related to improved adherence to controller therapy [127]*.* To reduce inflammation in the lungs, such as in patients with asthma, therapeutic effects can be achieved with low doses of ICSs, which are associated with minimal detectable systemic effects. Immunosuppression at high doses of ICSs is weighted toward the lungs, but moderate systemic immunosuppression could also be expected because of its dual local and systemic bioactivity [128]. Delivering corticosteroids directly to the distal airways and alveoli by inhalation could effectively reduce inflammation in the lungs with fewer systemic side effects. Apart from their anti-inflammatory effects, some ICSs have been found to have antiviral effects. In vitro, corticosteroids inhibit rhinovirus and respiratory syncytial virus-induced cytokine release [129], but the timing of exposure to ICSs seems important with pre-treatment being less effective than administration at the time of infection [130]. Ciclesonide and mometasone suppressed the replication of SARS-CoV-2 and MERS-CoV in vitro, whereas dexamethasone, cortisone, prednisolone, and fluticasone did not exert antiviral effects [131]*.* In addition, an in vitro experimental study has shown that glycopyrronium, formoterol, and a combination of glycopyrronium, formoterol, and budesonide can reduce coronavirus HCoV-229E replication, partly by inhibiting receptor expression and/or endosomal function [132]. Considering the difference in the features of the viruses, the results for coronavirus HCoV-229E should be interpreted cautiously [133]*.* Many clinical trials utilizing ICSs for COVID-19 have been registered on ClinicalTrials.gov: four trials for ciclesonide and four for budesonide (one including formoterol). Further studies are needed to investigate the possible positive effect of ICSs on COVID-19 pneumonia as previously shown with dexamethasone in the RECOVERY trial [134]. A recent meta-analysis revealed no significant difference in the risk for the development of a severe or fatal course of COVID-19 with preadmission use of ICSs in patients with COVID-19 relative to non-use of ICSs [135]. These findings assured the safety of continued use of ICSs during the COVID-19 pandemic. Understanding the basis of differences in susceptibility to severe COVID-19 between asthmatic and non-asthmatic populations may ultimately offer important insights into therapeutically exploitable targets to reduce the overall burden of COVID-19. There are no current data that support or recommend a step-down of current treatments of patients. ICSs undoubtedly decrease the rate of exacerbations in asthmatic patients. If people with stable asthma stop or reduce their ICSs inappropriately in response to concerns about immunosuppression and worries about developing COVID-19, they may be at significant risk of experiencing exacerbation. The European Forum for Research and Education in Allergy and Airway Diseases (EUFOREA) concluded that proper treatment of allergic rhinitis and allergic asthma is important, and topical corticosteroids can be used in such cases [136]*.* In hospitals, the use of metered-dose inhalers (MDIs) and dry powder inhalers (DPI) is preferred to nebulizers if patients can perform the breathing maneuvers. If a nebulizer is used, a high-flow nasal cannula is preferable to a face mask and a mouthpiece should be used with a jet or mesh nebulizer, and viral filters or one-way valves should be attached to nebulizers to minimize the release of aerosols [126].

In conclusion, clinicians should be aware that there is no evidence to support the withdrawal of ICSs in patients treated with these drugs, and to do so is likely to be harmful. Patients with asthma who are stable while using ICSs should continue their treatment. If there is uncertainty about the diagnosis, physicians should be more careful about initiating ICSs or ICS/long-acting β-agonist in patients without clear objective evidence of asthma. Similarly, there is no evidence to suggest a change in the recommendation for asthma patients to increase the dose of ICSs at the onset of an exacerbation. Based on the above, the indications contained in the GINA document about asthma and COVID-19 are certainly acceptable [137]: a) advise patients to continue taking their prescribed asthma medications, particularly ICSs; b) make sure that all patients have a written asthma action plan; c) advising them to: increase controller and reliever medication when asthma worsens; d) avoid nebulizers where possible, to reduce the risk of spreading the virus; e) pressurized MDI via a spacer is preferred except for life-threatening exacerbations, and f) add a mouthpiece or mask to the spacer if required.

### Biological therapies and COVID-19

Managing patients with severe asthma during the coronavirus pandemic and COVID-19 is a challenge. Unless relevant data during the pandemic is going to emerge soon, changing our understanding on the safety of biologic therapies, clinicians must follow the recommendations of current evidence-based guidelines for preventing asthma loss of control and exacerbations. Moreover, in the absence of data that would indicate any potential harm, the current advice is to continue the administration of biological therapies during the COVID-19 pandemic in patients with asthma for whom such therapies are indicated and have been effective [138]*.* For patients with severe asthma infected by SARS-CoV-2, the decision to maintain or postpone biological therapy until the patient recovers should be a case-by-case based decision supported by a multidisciplinary team [126]. Biologicals are indicated for severe asthma therapy in patients that are not controlled adequately with other treatments. The three anti-IL5/IL5r have their major effect in targeting eosinophils by producing either a reduction or depletion of tissue and peripheral blood eosinophils. The other two biologics (anti-IgE and anti-IL4/ IL13) have their primary effects by inhibiting Type 2 immunity. Thus, the primary question could be raised whether eosinophils and/or Type 2 mechanism immunomodulation have any role in modifying susceptibility, severity, immunity, or resistance to SARS-COV2 infection. Although the role of eosinophils in COVID-19 disease has yet to be elucidated, it has been shown that this viral infection can be associated with profound eosinopenia and that persistent eosinopenia may be associated with clinical deterioration and increased risk of mortality [116]. Several possible explanations for eosinopenia related to COVID-19 have been proposed: decreased eosinophilopoiesis, eosinophil apoptosis induced by type 1 IFN released during the acute infection, and increased eosinophil migration and retention within inflamed tissues [139]. There have been a few case reports in the literature of patients with COVID-19 who were on treatment with either omalizumab, mepolizumab, benralizumab, or dupilumab, but all had a favorable clinical course [140–146].

A multicenter Italian survey evaluated 473 consecutive patients with severe asthma treated with biological therapy (mepolizumab (*n* = 200), omalizumab (*n* = 145); benralizumab (*n* = 124); dupilumab (*n* = 4) [72]*.* Four of these patients contracted COVID-19 (3 were on omalizumab therapy and 1 on benralizumab); all of them were atopic patients. In contrast, no cases of COVID-19 were observed in the 200 patients treated with mepolizumab or the 4 patients treated with dupilumab. The four confirmed COVID-19 cases displayed good control of asthma symptoms before SARS-CoV-2 infection, without asthma exacerbations during the last 3 months before the illness. Three of them paused the therapy during illness. Two SARS-CoV-2 infected patients experienced a mild COVID-19, while two patients required admission to the ICU for severe and critical illness, respectively. All of them clinically recovered. Another observational study was carried out to evaluate the occurrence of COVID-19 in adult patients with severe asthma, based on data from the Belgian Severe Asthma Registry (BSAR), and to assess whether patients with severe asthma using biologics present an increased risk of severe COVID-19 compared to those who do not use these medications [68]*.* In this cohort of severe asthma patients, a small number of COVID-19 cases was found; none resulted in death or a very severe disease course. Of 676 participants, only 14 patients were identified with COVID-19 infection confirmed by either PCR and/or specific IgG. Of these 14 patients, only 5 had been hospitalized (with a hospital stay ranging from 2 to 8 days). None presented with a severe asthma exacerbation or required treatment with systemic corticosteroids, admission to the ICU, non-invasive ventilation, mechanical ventilation, or extracorporeal membrane oxygenation, and no deaths occurred. However, not all studies published so far are consistent with the previous one. Indeed, in a recent prospective ongoing survey from 15 hospitals of the Dutch Severe Asthma Registry (RAPSODI), 9 out of 634 (1.4%) severe asthma patients who received biologic therapy were diagnosed with COVID-19. Seven (1.1%) required hospitalization for oxygen therapy, 5 of whom were admitted to the ICU for intubation and mechanical ventilation. One patient died (0.16%). All intubated patients had ≥1 co-morbidity. Odds (95%CI) for COVID-19-related hospitalization and intubations were 14 (6.6–29.5) and 41 (16.9–98.5) times higher, respectively, compared to the Dutch population.

Although more data will need to be obtained in the future, the evidence currently available encourages the continuation of maintenance therapy and biologic treatment of patients with asthma in the context of this pandemic in non-infected individuals. A practical clinical algorithm has also recently been proposed on the use of biologicals for the treatment of allergic diseases in the context of COVID-19. Non-infected patients on biologicals for the treatment of asthma, AD, CRSwNP, or CSU should continue their biologicals targeting type-2 inflammation via self-application. In the case of an active SARS-CoV-2 infection and moderate-to-severe COVID-19, biological treatment needs to be stopped until clinical recovery and SARS-CoV-2 negativity are established. Thereafter, treatment with biologicals can be reinitiated [147]*.* Furthermore, maintenance of add-on therapy and a constant assessment of disease control, apart from acute management, are required. A consensus-based ad hoc expert panel of allergy/immunology specialists from the USA and Canada recommends continuing administration of biologicals in patients with proven efficacy and converting the patient to a prefilled syringe for potential home administration if this is available, otherwise in-office application is possible with a plan to transition to home administration [148]*.* Therefore, if available, it is recommended to prefer a formulation for self-application and to offer telemedical monitoring.

### Allergen immunotherapy and COVID-19

Regarding allergen immunotherapy (AIT), this biological therapy has been used in allergic diseases for more than 100 years, and many new therapeutic advances have been introduced in recent years [149]*.* The AIT approach to patients with respiratory allergies may provide a possible theoretical advantage to patients during the COVID-19 pandemic and can be continued if patients are not diagnosed with COVID-19. An essential part of the complex mechanism of action of AIT is the generation and maintenance of functional allergen-specific regulatory T (Treg) cells and regulatory B (Breg) cells [150]. Treg cells play a role in preventing cytokine storm and limiting tissue damage [151, 152]*.* It appears therefore conceivable that the immune tolerance induced by AIT might have a putative protective role in severe COVID-19 patients with cytokine storm. Furthermore, starting AIT in eligible patients, the sublingual route of administration (SLIT) is preferred to minimize in-person encounters for subcutaneous injections (SCIT); indeed, SLIT can be administered at home, thus avoiding the need to travel to or stay in an allergy clinic or doctor’s office, which would be associated with a risk of infection [153, 154].

Finally, interrupting AIT is cautiously recommended in patients diagnosed with COVID-19, or suspected of SARS-CoV-2 infection, or symptomatic patients with exposure or contact with SARS-CoV-2 positive individuals, or patients with positive SARS-CoV-2 RT-PCR test results [155]*.*

## Conclusions

Based on our review of the available literature on asthma and COVID-19, asthma does not confer an increased susceptibility to SARS CoV-2 infection or a worst clinical course in infected patients. Asthma per se does not appear to be a risk factor for COVID-19 overall; however, its actual contribution to the risk may depend on the presence of other environmental and behavioral factors (i.e. smoking, comorbidities) the type and severity of asthma (e.g. non-type 2 asthma phenotypes, uncontrolled asthma), asthma treatments and adherence to therapy (scarce adherence).

Asthma International Guidelines recommend complying with the basic measures of protection against COVID-19 and continuing asthma therapies including ICSs and biological agents in asthmatics during the COVID-19 pandemic. Maintaining current therapy with controller medications, including biological agents, is recommended in all patients with asthma, since the ongoing use of ICSs does not increase the risk of hospitalization in asthmatics with concomitant COVID-19 infection. Eventually, the decision to continue or postpone biologic therapy in patients already infected with SARS-CoV-2 should be individualized.

Further clinical and experimental studies will be necessary to confirm these preliminary data against a “dangerous liaison” between COVID-19 and asthma.

## Data Availability

N/A

## Abbreviations

ACO: Asthma-COPD overlap
ACE2: Angiotensin-converting enzyme2
AIT: Allergen immunotherapy
ARDS: Acute respiratory distress syndrome
COPD: Chronic obstructive pulmonary disease
COVID-19: Coronavirus disease 2019
FeNO: Fractional exhaled nitric oxide
ICAM-1: Intercellular adhesion molecule 1
ILC: Innate lymphoid cells
ICU: Intensive care unit
ICSs: Inhaled corticosteroids
IFN: Interferons
IL: Interleukin
IOS: Impulse oscillometry
ISARIC: International severe acute respiratory and emerging infections consortium
pDCs: Plasmacytoid dendritic cells
PFT: Pulmonary function testing
TSLP: Thymic stromal lymphopoietin
WHO: World health organization
